# Breakfast Consumption in French Children, Adolescents, and Adults: A Nationally Representative Cross-Sectional Survey Examined in the Context of the International Breakfast Research Initiative

**DOI:** 10.3390/nu10081056

**Published:** 2018-08-09

**Authors:** France Bellisle, Pascale Hébel, Aurée Salmon-Legagneur, Florent Vieux

**Affiliations:** 1 Nutri Psy Consult, 91 Santé, 75013 Paris, France; 2 CREDOC (Centre de Recherche pour l’Etude et l’Observation des Conditions de Vie), 142 rue du Chevaleret, 75013 Paris, France; hebel@credoc.fr (P.H.); salmonlegagneur@credoc.fr (A.S.-L.); 3 MS-Nutrition, 27 boulevard Jean-Moulin, 13005 Marseille, France; florent.vieux@ms-nutrition.com

**Keywords:** breakfast, diet quality, sugar, dietary survey, children, adults

## Abstract

This study examines the consumption of breakfast on the basis of a 7-day dietary record (Comportements et Consommations Alimentaires en France 2012–2013) in a representative sample of French children (*n* = 426), adolescents (*n* = 250), and adults (*n* = 1045). A large majority of the participants were regular consumers of breakfast (5–7 times per week). Breakfast accounted for 17.6% of total daily energy (339.4 kcal). Breakfast was rich in carbohydrates (24% of total daily intake) and simple sugars (31% of total daily intake). Relative to its contribution in daily energy intake, breakfast contributed higher proportions in the daily intake of many vitamins (B, C), and minerals (calcium, iron, iodine, manganese, phosphorus, potassium, magnesium). The main foods/beverages contributing to breakfast changed with age, with increasing contributions of non-wholegrain “bread and toasts” and “fruits”, and a decreasing contribution of milk. Better quality of the diet, as measured by tertiles of the Nutrient Rich Food Index 9.3, was associated with higher intakes of cereal products (bread and breakfast cereals, particularly wholegrain), dairy (milk, fresh dairy), and fruit at breakfast. In conclusion, breakfast is regularly consumed in France and contributes significantly to diet quality but could be improved in terms of content in fiber and protein.

## 1. Introduction

Breakfast is often presented as the “most important meal of the day”. Two recent scientific reviews have addressed many aspects of this notion [1,2]: nutritional, with possible impact on body weight control, physiological, psychological, social, cultural. One critical aspect is the fact that this early meal breaks the fast after the overnight hours and provides precious nutrients that will allow the individual to perform morning tasks with optimal efficiency [2]. The recent recognition of the importance of circadian rhythms that modulate biological functions at specific times of day [3] reinforces the interest of breakfast as a singular nutritional event [1].

Breakfast regularity and composition have been presented as critical to many health benefits. For example, the failure to have breakfast correlated with a 27% increase in heart disease in American men [4]. Having versus skipping breakfast favours more stable afternoon and evening glycemia [5]. Breakfast consumption has been shown to enhance cognitive function in children [6] and adults [7] during the morning hours, although non-significant results have also been reported [8].

Breakfast consumers around the world typically have lower BMI values compared to non-consumers [9,10,11,12,13,14], suggesting that the consumption of breakfast facilitates body weight control. In America, breakfast skipping increased over the years in parallel with the rise in obesity [15]. In recent years, however, the causal influence of breakfast consumption on body weight control has been questioned based on the non-significant effects obtained in randomized controlled trials [9,16,17,18].

Breakfast has been consistently reported to contribute to diet quality in various parts of the world among which Mexico [19], Australia [20], Europe [21,22]. Breakfast in many parts of the world has a typical menu, different from other eating occasions [1], which increases the diversity of the food sources in the diet. Policy makers generally recognize a healthy breakfast as being important for good nutrition and health [23] and national recommendations often include guidance about the frequency and content of breakfast. In France, the National Plan for Nutrition and Health (PNNS) [24] recommends children and adults to have a regular breakfast composed of a cereal product (preferably wholegrain), a fruit (preferably fresh whole fruit but 100% pure fruit juice or pureed fruit are also acceptable) and one dairy product. This meal is explicitly recommended to break the fast after the overnight hours and contribute a substantial proportion of the daily nutrients. 

The International Breakfast Research Initiative (IBRI) was launched in 2016 to examine breakfast consumption and recommendations in different countries (Canada, Denmark, France, Spain, United Kingdom, United States of America) [23]. Its objectives are to identify the frequency of breakfast consumption, to assess its contribution to the overall quality of the diet, and possibly to provide evidence-based quantitative advice to policy makers in order to improve local recommendations. The present paper addresses breakfast consumption in France in the context of the IBRI. We used the nationally representative survey of food intake in French children and adults (the “Comportements et Consommations Alimentaires en France”, CCAF survey) carried out by the Centre de Recherche pour l’Etude et l’Observation des Conditions de Vie (CREDOC) as a basis for assessing the consumption of breakfast in the French population. 

The main aim of this study is to quantify breakfast consumption in the French population and to analyse its nutritional contribution to the diet, its correlation with the body adiposity status and various aspects of the lifestyle. This analysis should identify how and in whom breakfast consumption can be improved in the French population and provide an objective basis for targeted recommendations. For comparison with previous research in the French population (e.g., consumption of wholegrain foods [25]) and with international data, a special emphasis was put on the consumption of wholegrain foods at breakfast and the use of dietary supplements.

## 2. Materials and Methods

The analyses conducted in this paper followed a harmonized approach defined within the International Breakfast Research Consortium [23].

The data were extracted from a nationally representative survey of 7-day food intake in French adults, adolescents, and children in 2012–2013 (the CCAF survey). CCAF surveys are cross-sectional studies carried out at regular intervals in the French population. They collect data on anthropometry, socio-economic status, lifestyle, and diet in representative samples of French consumers characterised by age, sex, body adiposity status, profession and several lifestyle variables, such as smoking and daily screen-watching time.

In the CCAF survey methodology, participants are asked to report their intake at the eating event they themselves identify as “breakfast”. The “breakfasts” reported by respondents in the 2012–2013 CCAF were analysed for frequency, social and physical circumstances, food and nutrient content, and contribution to the quality of the diet.

### 2.1. Population

The details of participant recruitment are consistent with the CREDOC methodology, as described in previous publications [26]. The survey was carried out between November 2012 and July 2013 in a nationally representative sample of 1262 households, in which all individuals over 3 years of age were interviewed. An extra national sample of households from which only children were invited to participate were randomly recruited in order to have sufficient numbers of children and adolescents (3–18 year-olds) in the sample (*n* = 862). Age, socio-economic status (based on occupation of head of household as classified by the National Institute of Statistics), geographical region, town size and household size were taken into consideration in the quota sampling method. 

The sample was subdivided into four age groups: children (6–12 years-old), adolescents (13–17 years-old), younger adults (18–54 years-old), and older adults (55 years-old and above). Data for children between 3 and 6 years of age were not included in the present analyses.

For each participant, self-reported height, weight, and time spent on physical activity and sedentary (screen watching) behaviour were recorded in face-to-face interviews with experienced members of staff. Children reported in the presence of parents or caregivers. The participants then completed a seven-day food intake record, and reported the types and amounts of all foods and beverages consumed. Dietary intake was reported with the help of parents or caregivers for all children aged 9 years or less and for older children who needed assistance. 

The energy intake reported by the participants was compared with the estimated energy requirements (at least 1.55 times the metabolic rate), according to Schofield’s equation [27]. Adults were considered under-reporters and excluded if their reported energy intake was lower than the estimated energy requirements. Children were considered under-reporters and excluded if their total energy intake divided by estimated basal metabolic rate was lower than 0.5.

To control for seasonal differences in intake, the survey was carried out in four successive phases (November–December, January–March, April–mid-June, and mid-June–July), during which approximately a quarter of the participants were included. The CCAF study was approved by the French Commission Nationale de l’Informatique et des Libertés (CNIL). All participants provided informed consent. For children and adolescents, parents’ consent was obtained.

### 2.2. Determination of Body Adiposity Status

BMI was calculated as weight/height^2^ (kg/m^2^). In adults, BMI values between 18.5 and 25 kg/m^2^ were considered to represent normal body adiposity status. Overweight was defined as values between 25 and 30 kg/m^2^ and obesity as BMI values of 30 kg/m^2^ and above. Underweight corresponded to BMI values under 18.5 kg/m^2^. In children, the body adiposity status was defined on the basis of growth curves and cut-off values for age presented by Cole et al. [28].

### 2.3. Dietary Intake Data

The participants reported the types and amounts of all foods and beverages consumed over seven consecutive days. A “diary” was provided to each participant, to be completed either in paper or electronic format, as the participant preferred. Experienced staff visited each household and explained participants how to complete the diary before the start of the reported week. For each eating occasion during the week, all intakes of foods and beverages had to be reported. The diary had separate pages for the various eating occasions during each day, including main meals (“breakfast”, “lunch”, and dinner”) and the various possible eating occasions before or after each of these main meals. To facilitate the reporting of portions, participants were provided with the validated SUVIMAX portion size atlas [29], showing photographs of various common foods and beverages in different portion sizes. The use of this instrument was explained by the staff to each respondent before the beginning of the reported week. During the week, participants could obtain answers to any question by telephone from the staff. At the end of the week, the members of staff again visited the households, reviewed all completed diaries with the participants and clarified any ambiguity in the declared data.

The energy and nutrient contents of consumed foods and drinks were obtained from the CIQUAL French food composition table [30]. The CIQUAL table was classified into 42 food groups. The contents in added sugars of the CIQUAL foods and beverages were estimated using the systematic methodology proposed by Louie et al. [31] in which “added sugars” are defined as refined sugars added during cooking or manufacturing.

Intakes were computed for the whole day and for individual eating occasions (main meals, including breakfast, and snacks). In addition, the circumstances of intake were reported by the participants, including time of day, day of the week, place and context of consumption.

### 2.4. Assessment of Breakfast Consumption

The intake diary included a specific “Breakfast” page where participants reported the food composition and circumstances (time, location, etc.) of the eating occasion they themselves identified as breakfast. The “Breakfast” page was taken to represent breakfast on this particular day, with no minimum energy content.

Frequent consumers of breakfast were defined as participants reporting 5–7 breakfasts a week; irregular breakfast consumers were respondents with 2–4 breakfasts over 7 days; “breakfast skippers” were individuals who reported 0–1 breakfast over the week.

Participants were required to report the time and location of breakfast consumption, the company who shared the meal (number of persons and type of relationship), what else they were doing at the time of breakfast (nothing, watching a screen, or doing something else: reading, listening to the radio, etc.) Breakfast intake data were analysed according to age, sex, body adiposity status, socio-economic status and other aspects of lifestyle (for example smoking, physical activity or screen watching time).

### 2.5. Specific Assessment of Wholegrain Consumption

The CIQUAL cereal food groups (“Sweet crackers and biscuits”, “Breakfast cereals”, and “Breads and toasts”) were checked for the presence of wholegrain (from wheat, oats, barley, rice, maize, rye, buckwheat, quinoa, bulgur, millet, spelt and amaranth). The wholegrain content of foods was obtained from brand information and quantitative nutrient declarations (QUIDS) on food labels. QUIDs were obtained from MINTEL (a market research database) [32], manufacturer’s websites, or online shopping websites. If brand or QUID information was not available, then details of the wholegrain content of similar products were used (products of the same brand with available QUIDs, or similar ingredient list, or similar name and description). Total wholegrain consumption (g/day) was computed as the total intake from the various food sources.

### 2.6. Assessment of Use of Dietary Supplements

Dietary supplement use was assessed directly by the answer to the question: “Do you regularly consume dietary supplements or vitamins or minerals in specific forms (e.g., tablets, capsules, powder, vial…)?” This information was obtained only from adults (18 years and older).

### 2.7. Assessment of Nutrient Density

Nutrient density of the total daily diet was assessed using the Nutrient Rich Foods (NRF) index [33,34,35]. Initially developed to score nutrient density of individual foods, the present variant was used to assess to assess the nutritional adequacy of the diet [23]. The NRF9.3 score is based on 9 qualifying nutrients and 3 disqualifying nutrients. The qualifying nutrients are protein, fiber, vitamin A, vitamin C, vitamin D, calcium, iron, potassium, and magnesium. The disqualifying nutrients are saturated fat, added sugar, and sodium. The overall daily intake of each nutrient for each subject was normalised for 2000 kcal and expressed as a percentage of the reference daily intake used for nutritional labelling the Europe [36] (except fibers and free sugars for which WHO recommendations [37] were used, and SFA for which the French recommendation [38] was applied). For qualifying nutrients, each percentage of the reference daily intake was truncated at 100 so that a high intake of one nutrient could not compensate for the low intake of others. For disqualifying ones, only the share in excess of the recommended amount was considered. The NRF9.3 algorithm is the sum of the percent daily values for the 9 qualifying nutrients minus the sum of daily values of 3 disqualifying nutrients. The sample was then stratified by NRF tertiles.

### 2.8. Assessment of Daily Screen Watching Time

Screen watching was used as a proxy for the level of sedentary behaviour. The time spent watching various screens (television, computer, video games, etc.) was reported by the participants at the time of the face to face interview with the member of staff. Two levels (“low” and “high”) were arbitrarily defined for screen watching time in adults (more or less than 3 h/day) and in children and adolescents (more or less than 2 h/day).

### 2.9. Assessment of Daily Physical Activity in Adults

The time spent on various physical activities (household activities, gardening, sports, etc.) was reported by the participants at the time of the face to face interview with the member of staff. The total time spent daily was computed. For adults, two levels were arbitrarily defined (low = less than 2 h/day; high = 2 h/day or more) in accordance with previous CREDOC studies. Because of the large differences in types and intensity of physical activity from childhood to late adolescence, no such index was computed in the children-adolescent sample.

### 2.10. Statistical Analyses

The SAS 9.4 software (SAS Institute, Inc., Cary, NY, USA) was used for statistical analyses and for database management. Differences between proportions were tested using X^2^ tests. Differences in quantitative variables (such as intakes) were tested using ANCOVA, adjusted as appropriate. Multiple comparisons were addressed using Tukey’s multiple comparison test for continuous variables and Bonferroni-Holm test for categorical variables. Continuous data are reported as means and standard deviations. The confidence level for calculated confidence intervals was 95%. The statistical significance level was set at *p*-value < 0.05.

## 3. Results

Paper (54%) versus online (46%) reports showed no significant differences in terms of energy intake and consumption of most food categories, macro- and micronutrients. Examination of the breakfast data revealed a few instances of outlier values (excessively long breakfasts, or over 3 SD above the mean energy intake, adjusted for age) suggesting faulty reporting of the termination of the meal. All breakfasts over 1185.9 kcal (>3 SDs) were then excluded from the data set. These instances accounted for less than 1% of the total number of reported breakfasts, leaving 11053 breakfasts into the final analysis. In only 46 instances, participants reported one intake event before the declared “breakfast”.

From the 3122 participants initially recruited in the CCAF 2013 study, 1716 were included in the study after exclusion of under-reporters. There were no differences in gender distribution or education level between the included and excluded participants. The proportion of overweight and obese individuals (BMI over 25 kg/m^2^) among the excluded participants was greater than among the included participants (*p* < 0.05).

**Figure 1:**
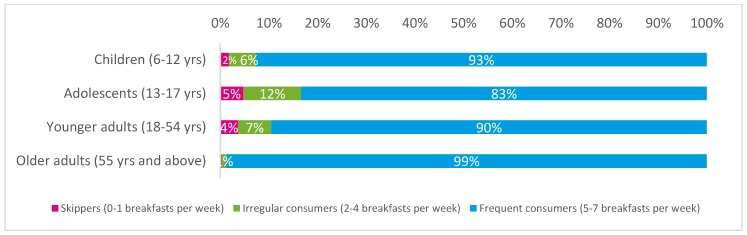
Proportion of skippers, irregular and frequent consumers, per age group.

The final sample included 426 children, 250 adolescents, 595 younger adults, and 445 older adults. Figure 1 shows the distribution of number of breakfasts reported in the four age groups. Most participants in all age groups (91% overall) were frequent consumers of breakfast (5 breakfasts or more over 7 days). The proportions of irregular consumers and skippers were very low. Adolescents had the highest proportions of irregular consumers (12%) and skippers (5%) (X^2^ = 127.5; *p* < 0.0001). The low number of irregular consumers of breakfast and skippers in all age groups makes comparisons with the rest of the sample statistically inapplicable.

Breakfast consumption was as frequent on week days and week-end days. Children, adolescents, and younger adults had breakfast around 7h–7h30; older adults reported breakfast around 8h–8h30. Breakfasts were consumed at home by over 93% of respondents of all age groups, socio-economic status, or body adiposity status. Breakfast was consumed alone in just over 50% of adolescents and adults and 24% of children. Breakfast was consumed in the company of family members in 70% of children, 40% of adolescents , and 38% of adults. Eating breakfast alone was more frequent in obese (58%) and overweight (53%) respondents than in normal weight (48%) and underweight (42%) participants (X^2^ = 108.1; *p* = 0.0001).

Most participants reported doing nothing else at breakfast time (51% females; 53% males), whereas around 30% had breakfast while looking at a screen (TV, video games, etc.). In respondents with a high daily screen time, this proportion was significantly higher (35%) than in respondents with a low level of daily screen time (25%) (X^2^ = 155.6, *p* = 0 < 0.0001). Screen watching at breakfast time was negatively associated with age (from 39% in children to 20% in older adults) (X^2^ = 998.7; *p* < 0.0001). Larger proportions of normal weight (54%) and underweight (56%) participants did nothing else at breakfast time, versus overweight (49%) and obese (41%) respondents (X^2^ = 238.3; *p* < 0.0001).

Table 1 presents energy and macronutrient intakes at breakfast and for the whole day in each age group. Table 2 presents micronutrient intakes. Breakfast contributed 339.4 ± 186.5 kcal on average for the whole population, representing 17.6% of the total daily energy intake. Breakfast was relatively rich in carbohydrates (24% of total daily intake) and simple sugars (31% of total daily intake). While the energy content of breakfast did not vary significantly according to age group, sugar content peaked at adolescence and then decreased with age (Table 1). Very few significant age differences appeared in the intake of micro-nutrients at breakfast: only copper and selenium increased with age (Table 2).

**Figure 2:**
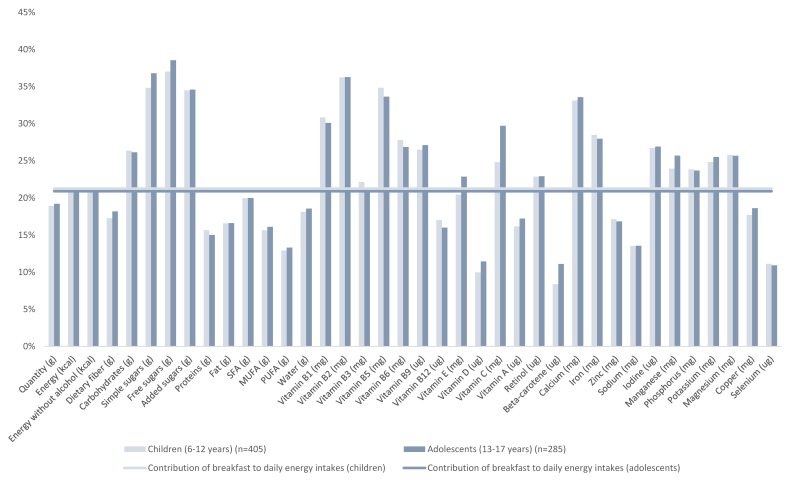
Contribution of breakfast to daily energy and nutrient intakes, for children and adolescents.

**Figure 3:**
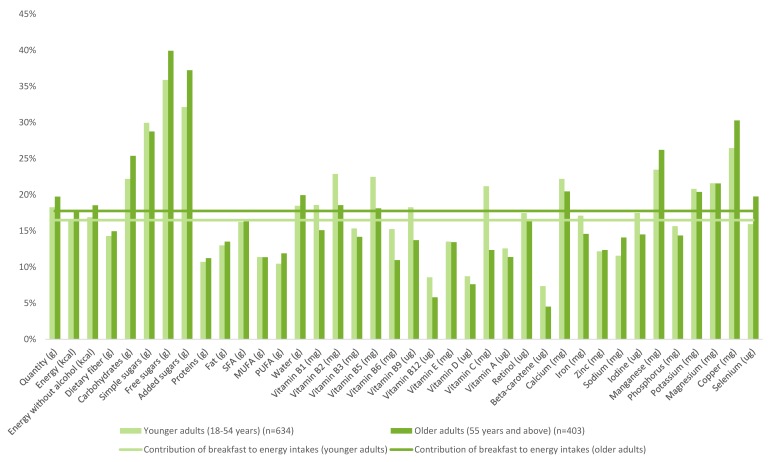
Contribution of breakfast to daily energy and nutrient intakes, for younger and older adults.

The percent contribution of breakfast to daily nutrient intakes in children and adolescents is illustrated in Figure 2; Figure 3 displays corresponding data for adults. In children and adolescents, breakfast contributed a higher proportion of many vitamins (B and C) and minerals (calcium, iron, iodine, manganese, phosphorus, potassium, and magnesium) than energy to the daily diet. In adults (Figure 3), breakfast contributed less than 20% of daily energy but provided higher proportions of calcium, manganese, potassium, magnesium and copper. 

Only 14.5% of the adult sample (*n* = 151) reported using dietary supplements at least once during the 7 days of the food record. Therefore, this low intake was not included in the computations of daily nutrient intake.

### 3.1. Food Composition of Breakfasts

Table A1 (see Appendix A) displays the contribution of the various food (in grams) and beverage (in mL) groups to breakfast in children, adolescents, younger and older adults. The contributions of breads and toasts (wholegrain or not) increased with age while breakfast cereals (wholegrain or not) decreased. Consumption of fruits and dairy products were highest in older adults. Children + adolescents drank more milk and juice than adults did, while adults drank hot beverages mainly. The intake of wholegrain products (cereals, crackers, biscuits, bread, toasts) at breakfast was below 10 g in all age groups .

Table 3 gives the contribution of food groups to the intake of nutrients at breakfast. For the sake of clarity, the data are presented for two age groups: below or above the age of 18 years. Cereal products (bread, breakfast cereals, viennoiseries), fruit (whole and fruit juice), and dairy (milk and fresh dairy products) were important contributors in both age categories, although the food composition of breakfasts changed with age (Table A1).

### 3.2. NRF9.3 Data

The NRF 9.3 algorithm was used to obtain a score of diet quality in all participants. The values obtained allowed the identification of three tertiles of dietary quality in children + adolescents, and in adults. In children + adolescents, the NRF 9.3 scores were significantly affected by socio economic parameters, such as the education and profession of the head of household; in adults the NRF 9.3 scores were significantly affected by age and by socio economic variables (Table A2). Except for the protein and sodium subscores in children + adolescents, the three tertiles of diet quality scores were highly significantly different (Table A3).

Table 4 presents nutrient intakes at breakfast across tertiles of the NRF 9.3 scores. Statistical comparisons between tertiles were adjusted for socio economic parameters (education and profession of head of household) in children and for these same socio economic variables plus age in adults. While energy intake at breakfast was not different across tertiles, several nutrients significantly increased (fibre, protein, B vitamins, Vitamin C, calcium, iron, zinc, potassium, magnesium; in children only Vitamin A, retinol, sodium) or decreased (added sugar) as dietary quality scores increased.

The breakfast food choices across tertiles of NRF 9.3 are presented in Table 5 for both age categories (below or over 18 years). Participants in the top tertiles of daily dietary quality, as defined by the NRF 9.3 score, ingested significantly more milk, bread and toasts, breakfast cereals than participants in the lowest tertiles. Adults of the highest NRF 9.3 tertiles also had more wholegrain bread and toasts, wholegrain breakfast cereals, fresh dairy products, and fruits, but less viennoiseries than adults of the lower tertiles.

## 4. Discussion

The International Breakfast Research Initiative [23] proposes a novel and harmonised approach to the study of the nutritional impact of breakfast involving national dietary survey data from several countries. The present paper is the French contribution to this initiative.

The results of the present study confirmed that breakfast is a very regular meal in France, ingested by over 90% of all age groups, at home, in the company of family members about half of the time, and while doing nothing else for most respondents but with a frequent report of meal-time screen watching in younger respondents. The French breakfast appears a regular source of many nutrients (over 20% of the daily input of many vitamins and minerals, particularly in children and adolescents) for an energy load representing 17.6% of the daily total. The present observations agree with the recently published INCA 3 survey, carried out in 2014–2015 in a larger representative sample of the French population (*N* = 5855), but deriving intake data from three 24 h food reports [39]. The INCA 3 data confirmed that breakfast is a carbohydrate-rich meal consumed by a majority of the population, representing 18.6% of daily energy in children (11–17 years-old) and 17.2% in adults (18–79 years old).

One recurrent problem in breakfast studies is that of definition. A definition of breakfast has been proposed previously in the scientific literature: “Breakfast is the first meal of the day that breaks the fast after the longest period of sleep and is consumed within 2 to 3 h of waking; it is comprised of food or beverage from at least one food group, and may be consumed at any location.” [40]. In the present survey, breakfast was defined by the participants themselves who used a page of the food diary entitled “Breakfast”. Although the time of waking was not reported, the early consumption of “Breakfast” suggests that it was indeed consumed within 2–3 h of waking. In a large majority of cases (>99.5%), it was also the first reported eating event of the day. The question of the minimum amount of energy required to “break the fast” is an important one: while the consumption of a calorie-free beverage can be at times reported as “Breakfast”, it does not really break the overnight fast in the physiological sense. In many studies investigating the distribution of intake over the course of one day, a “meal” is defined as an eating event that represents at least 50 kcalories [41,42]. In the present survey, 11053 “Breakfasts” were examined. A small proportion of these contained less than 50 kcal (less than 2% in children and adolescents, 12% in younger adults and 6% in older adults). Given these low frequencies, it was decided not to fix a minimum limit of energy content in our analyses of “Breakfasts”.

Since most participants reported a high regularity of breakfast consumption, the low number of skippers made a statistical comparison of regular consumers versus skippers inappropriate. Irregular consumers were few and found mainly in the adolescent age group (12%). The observation of a lower regularity of breakfast intake in adolescents, compared to children and adults of the same populations, is a common finding (for example Alexy et al. 2010 in Germany [43]; Fayet-Moore et al. 2016 in Australia [20]). These recurrent observations suggest that the (relatively) lower frequency of breakfast in adolescents is a transient phenomenon that reverts to regular consumption later in life. 

Overweight and obese respondents in our sample reported having breakfast as frequently as normal weight peers, but they ate alone more frequently and more often tended to have another activity at breakfast time, particularly screen watching in younger people. Screen watching at the time of meals has often been found to increase the amount eaten in children and adults [44,45,46,47]. The causal links between these observations cannot be ascertained in this cross-sectional survey, but the data are compatible with the PNNS recommendation of making every meal a convivial shared moment [24].

The French PNNS presents a series of lifestyle recommendations for the French public of all ages [24]. Specifically, it recommends a regular 3-meals-a-day pattern, where people sit and stop their other activities to enjoy their meal, preferably in good company. According to the French guidelines, the first meal of the day should be composed of 2 or 3 foods (one cereal food, one dairy product, and one fruit) accompanied by one beverage. The cereal food should preferably be wholegrain, the dairy product can be a glass of milk, a yogurt or a piece of cheese, while the fruit can be fresh, canned or in the form of juice, without any added sugar. The present survey showed that French children frequently have milk (plus occasional fresh dairy products) at breakfast, and have various sorts of cereal products (bread, breakfast cereals, “viennoiseries”). Their consumption of fruit is not frequent (9%) although over half of them have fruit juice at breakfast. In French adults, the consumption of dairy and cereal foods at breakfast is also frequent, with a low frequency of fruit (14.3%) or fruit juice (33.1%) intake. Wholegrain bread and cereals remain rare choices on the breakfast table.

The NFR 9.3 index [33] was used to assess the nutritional quality of the diet and allowed tertiles of dietary quality to be identified in our samples of children and adolescents (up to 17 years of age) and adults (18 years and over). Breakfast composition was different between the highest and the lowest tertiles of dietary quality but its energy content was the same. Breakfasts of the highest tertiles of the population contributed more fiber and many vitamins and minerals for the same amount of energy. The NRF is a totally nutrient-based index but it was expressed in specific food choices at breakfast time: the highest tertiles consumed more milk, breads and toasts, breakfast cereals (notably wholegrain cereals); in addition the highest tertile of the adult population had more dairy products and fruit, but less “viennoiseries” than lower tertiles. The absolute level of intake of wholegrain products, however, remained quite low. These observations suggest that an increase in fiber content (via wholegrain foods) could be recommended to everyone, including persons in the highest tertile of NRF score. In individuals with lower diet quality, breakfast could be improved by the consumption of a protein-rich food (milk, fresh dairy, cheese, or other) and less added sugar.

The strengths of the present study include the seven-day food record method used to obtain intake data and the large nationally representative sample. A potential limitation of the present design is the fact that participants were recruited as members of households, which may have decreased the variability of dietary responses. A large proportion of the recruited participants had to be excluded as under-reporters, in spite of the high level of care provided to obtain good quality reporting of dietary intake. This is a clear limitation of the study that perhaps derives, at least partially, from the representative nature of the sample, with random recruitment among the French population. Some members of the households selected by the random recruitment process had sufficient motivation to accept to participate in the study but may not have had the optimal level of knowledge, understanding, or motivation required to deliver what was expected of them. Obese persons typically under-report food intake more than nonobese peers [48]. Although there is nothing exceptional in the present observation of more frequent under-reporting in overweight/obese participants, the exclusion of many overweight/obese under-reporters also possibly limits the sensitivity of the observations at the higher end of the body weight spectrum. The data are cross-sectional observations and thus do not allow any demonstration of causal effects.

## 5. Conclusions

The present observations confirm that breakfast is consumed regularly by most French children and adults. Breakfast is a CHO-rich meal, representing about 18% of the daily energy. While its content in free sugars is high, it also contributes significant proportions of several vitamins and minerals, particularly in children and adolescents. It could be improved in terms of its content of fiber and protein.

## Figures and Tables

**Table 1:** Energy and macronutrient intakes at breakfast and for the total day in the French population by age group.

	Total Population (*n* = 1727)				Children (6–12 years) (*n* = 405)				Adolescents (13–17 years) (*n* = 285)				Younger Adults (18–54 years) (*n* = 634)				Older Adults (55 years and above) (*n* = 403)				ANCOVA, Adjusted for Daily Total Energy Intake *p*-Value for Population Effect	
	Breakfast Intake		Daily Intake		Breakfast Intake		Daily Intake		Breakfast Intake		Daily Intake		Breakfast Intake		Daily Intake		Breakfast Intake		Daily Intake			
	Mean	SD	Mean	SD	Mean	SD	Mean	SD	Mean	SD	Mean	SD	Mean	SD	Mean	SD	Mean	SD	Mean	SD	Breakfast Intakes	Daily Intakes
Quantity (g)	430.2	195.7	2286.1	754.2	324.2 ^a^	108.9	1719.2 _a_	452.4	372.5 ^a^	142.2	1935.7 _b_	613.4	421.0 ^b^	206.3	2292.9 _c_	795.0	485.9 ^c^	173.1	2495.2 _d_	605.5	<0.0001	<0.0001
Energy (kcal)	339.4	186.4	1921.8	575.3	352.4	129.8	1656.4 _a_	452.7	403.1	175.6	1937.9 _a_	682.5	325.1	200.1	1948.6 _b_	595.1	343.6	160.4	1950.0 _c_	480.6	0.097	<0.0001
Energy without alcohol (kcal)	339.4	186.5	1865.3	556.6	352.4	129.8	1656.1 _a_	452.5	403.1	175.6	1934.7 _a_	683.8	325.0	200.1	1891.9 _a_	574.2	343.6	160.4	1861.7 _b_	458.8	0.097	0.015
Dietary fiber (g)	2.6	2.0	16.6	6.1	2.3	1.3	13.3 _a_	4.4	2.8	1.8	15.3 _a_	6.0	2.4	2.0	16.0 _b_	5.7	2.9	1.9	18.7 _c_	5.6	0.918	<0.0001
Carbohydrates (g)	52.6	30.3	217.0	72.3	54.4	20.7	206.5 _a_	61.3	61.5	28.4	237.5 _b_	94.7	50.1	32.5	219.2 _c_	74.4	54.2	26.7	212.0 _c_	60.9	0.274	0.014
Carbohydrates (% energy wa)	61.2	16.6	45.8	8.7	61.1 ^a^	11.3	49.2 _a_	6.8	60.4 ^a, c^	13.9	47.9 _b_	9.4	60.3 ^b,c^	18.2	45.2 _c_	9.5	62.8 ^d,e^	14.4	45.4 _d_	6.7	<0.0001	<0.0001
Simple sugars (g)	25.2	16.0	81.6	38.0	30.7 ^a^	12.3	90.0 _a_	32.2	34.5 ^a^	15.6	96.6 _a_	43.6	24.4 ^b^	17.0	81.2 _b_	41.5	21.9 ^b^	13.2	74.8 _c_	27.2	0.008	<0.0001
Free sugars (g)	17.6	12.6	49.3	31.3	21.0 ^a^	10.0	59.6 _a_	25.2	24.4 ^a^	13.6	67.0 _b_	37.8	17.4 ^b^	13.1	51.6 _c_	33.3	14.7 ^c^	10.4	36.7 _d_	19.7	0.032	<0.0001
Free sugars (% energy wa)	22.9	17.9	10.3	5.5	23.9	9.8	14.1 _a_	4.4	24.9	14.1	13.4 _b_	5.3	24.3	20.1	10.5 _c_	5.7	19.4	16.0	8.0 _d_	4.0	0.257	<0.0001
Added sugars (g)	13.8	10.7	42.9	28.4	16.6 ^a^	8.9	51.0 _a_	23.4	18.3 ^a^	12.0	56.4 _b_	32.0	13.4 ^b^	10.9	45.0 _c_	31.1	12.3 ^b^	9.4	32.9 _d_	18.4	0.019	<0.0001
Aded sugars (% energy wa)	18.3	16.7	9.0	5.0	18.4	7.2	12.0 _a_	4.0	17.5	8.6	11.3 _b_	4.8	19.1	18.7	9.1 _b,c_	5.2	16.8	15.7	7.2 _c_	3.7	0.152	<0.0001
Proteins (g)	9.3	5.9	81.5	26.0	10.5	4.4	67.1	19.3	11.9	6.0	80.5	30.0	8.9	6.6	83.0	26.7	9.1	4.7	84.1	22.5	0.286	0.140
Proteins (% energy wa)	11.8	10.0	17.4	3.9	11.7	3.4	16.2 _a,b_	3.0	11.6	4.0	16.4 _a_	3.9	11.9	11.2	17.3 _b_	4.2	12.0	10.3	18.1 _a,b_	3.0	0.908	<0.0001
Fat (g)	10.2	7.6	74.6	26.4	10.3	5.2	62.4 _a_	18.9	12.1	7.3	73.6 _a_	27.0	9.9	7.7	75.9 _a_	26.8	10.0	7.0	75.3 _b_	23.5	0.109	0.045
Fat (% energy wa)	25.5	12.4	35.3	7.2	26.2 ^a^	9.5	33.5 _a_	5.3	26.0 ^a^	10.8	33.6 _a_	7.3	25.5 ^a^	12.9	35.1 _a_	7.9	24.9 ^a^	11.6	36.2 _a_	5.9	0.010	<0.0001
SFA (g)	5.1	4.4	29.6	12.2	5.1	2.9	25.6 _a,c_	8.2	5.8	3.4	29.5 _a_	11.6	5.0	4.5	29.9 _a,c_	11.7	5.0	4.4	29.8 _b,c_	12.6	0.256	<0.0001
SFA (% energy wa)	12.5	7.4	14.0	3.6	13.1 ^a^	5.7	13.8 _a_	2.6	12.6 ^a^	5.5	13.5 _a_	3.4	12.6 ^a^	7.6	13.8 _a_	3.8	12.3 ^a^	7.5	14.2 _a_	3.3	0.011	<0.0001
MUFA (g)	2.9	2.2	24.6	9.2	3.2	1.7	20.6 _a_	6.5	3.9	2.6	24.4 _a_	9.5	2.8	2.2	25.1 _b_	9.5	2.7	1.8	24.7 _c_	7.8	0.058	0.018
PUFA (g)	1.1	1.0	9.6	4.7	0.9	0.6	7.3	3.0	1.1	0.9	8.7	4.1	1.0	0.9	9.6	4.4	1.2	1.1	10.2	4.8	0.619	0.452
Water (g)	351.8	177.7	1868.2	677.4	243.5 ^a^	89.1	1354.0 _a_	378.1	280.3 ^a^	113.7	1510.3 _a_	495.5	346.1 ^b^	185.7	1870.6 _b_	708.1	405.5 ^c^	160.5	2070.5 _b_	558.5	<0.0001	<0.0001

SFA: Saturated fatty acids; MUFA: Monounsaturated fatty acids; PUFA: Polyunsaturated fatty acids; wa: without alcohol; On a given line, different letters indicate significant differences between age groups according to the post-hoc test: superscripts for breakfast data comparisons, subscripts for total day data comparisons.

**Table 2:** Micro-nutrient intakes at breakfast and for the total day in the French population by age group.

	Total Population (*n* = 1727)				Children (6–12 years) (*n* = 405)				Adolescents (13–17 years) (*n* = 285)				Younger Adults (18–54 years) (*n* = 634)				Older Adults (55 years and above) (*n* = 403)				ANCOVA, Adjusted for Daily Total Energy Intake *p*-Value for Population Effect	
	Breakfast Intake		Daily Intake		Breakfast Intake		Daily Intake		Breakfast Intake		Daily Intake		Breakfast Intake		Daily Intake		Breakfast Intake		Daily Intake			
	Mean	SD	Mean	SD	Mean	SD	Mean	SD	Mean	SD	Mean	SD	Mean	SD	Mean	SD	Mean	SD	Mean	SD	Breakfast Intakes	Daily Intakes
Vitamin B1 (mg)	0.2	0.2	1.1	0.4	0.3	0.2	1.1 _a_	0.4	0.4	0.3	1.2 _a,c_	0.7	0.2	0.3	1.1 _b_	0.5	0.2	0.2	1.1 _b,c_	0.3	0.409	<0.0001
Vitamin B2 (mg)	0.4	0.4	1.6	0.6	0.6	0.3	1.5 _a_	0.5	0.6	0.5	1.7 _a,b_	0.9	0.4	0.4	1.5 _c_	0.7	0.3	0.2	1.6 _c,b_	0.5	0.360	<0.0001
Vitamin B3 (mg)	2.8	3.0	17.3	7.2	3.3	2.5	14.0 _a_	5.5	3.7	3.8	16.9 _a_	8.9	2.9	3.2	18.0 _b_	7.6	2.5	2.5	17.3 _b_	5.9	0.994	0.0007
Vitamin B5 (mg)	1.0	0.9	4.2	1.7	1.5	0.9	4.1 _a_	1.4	1.7	1.4	4.7 _a_	2.9	1.0	1.0	4.2 _b_	1.6	0.8	0.5	4.2 _a,b_	1.4	0.274	<0.0001
Vitamin B6 (mg)	0.3	0.3	1.6	0.6	0.4	0.3	1.5 _a_	0.5	0.5	0.5	1.7 _a_	1.0	0.3	0.3	1.6 _a,b_	0.6	0.2	0.2	1.6 _b_	0.5	0.368	<0.0001
Vitamin B9 (ug)	46.4	39.4	251.4	99.0	58.2	30.2	217.5 _a_	71.9	67.1	47.6	244.2 _a_	129.4	46.6	41.7	242.6 _a_	97.8	38.6	29.6	276.9 _b_	85.7	0.472	<0.0001
Vitamin B12 (ug)	0.4	0.4	5.4	5.3	0.6	0.3	3.7	2.3	0.6	0.5	4.4	4.2	0.4	0.5	4.9	3.7	0.3	0.3	6.7	6.6	0.239	0.532
Vitamin E (mg)	1.1	1.1	7.4	4.0	1.3	0.8	6.3	2.5	1.6	1.4	7.1	3.5	1.0	0.9	7.3	3.9	1.1	1.2	7.7	4.2	0.893	0.147
Vitamin D (ug)	0.2	0.3	2.4	1.7	0.2	0.2	1.7	1.0	0.2	0.4	2.1	1.6	0.2	0.4	2.5	1.9	0.2	0.2	2.6	1.4	0.819	0.602
Vitamin C (mg)	20.3	30.0	86.3	55.8	22.0	22.7	78.2 _a_	41.9	28.9	35.5	82.8 _a_	72.7	21.4	30.7	79.3 _b_	53.0	15.3	24.1	99.5 _c_	50.1	0.806	<0.0001
Vitamin A (ug)	78.2	80.8	824.4	863.3	70.3	43.2	551.0	415.7	79.6	53.4	615.9	684.6	75.3	84.6	751.6	676.4	82.3	77.5	1021.3	995.0	0.251	0.473
Retinol (ug)	63.9	65.4	563.4	841.5	59.7	38.3	343.2	361.4	66.4	48.9	402.8	609.2	59.4	61.4	506.9	687.0	70.3	73.5	705.8	974.1	0.341	0.503
Beta-carotene (ug)	129.1	337.4	2326.5	1870.0	114.3	185.6	1710.0 _a_	1265.0	143.6	252.3	1808.4 _a_	2145.3	134.9	365.8	2159.7 _a_	1811.0	107.6	226.3	2925.1 _b_	1645.6	0.170	0.001
Calcium (mg)	219.0	158.0	910.2	361.9	285.1	133.0	844.2 _a_	271.1	315.1	177.9	926.7 _a_	428.3	208.9	169.5	896.8 _b_	387.5	193.6	116.4	939.1 _a_	275.2	0.126	<0.0001
Iron (mg)	2.1	2.051	11.5	4.7	3.0	1.8	10.1	3.6	3.4	2.4	11.8	5.5	2.1	2.1	11.5	4.6	1.8	1.7	12.0	4.6	0.522	0.089
Zinc (mg)	1.2	0.9	9.6	3.5	1.3	0.7	7.8 _a_	2.7	1.5	1.0	9.3 _a,c_	4.3	1.2	1.0	9.6 _b,c_	3.4	1.2	0.9	10.0 _d_	3.2	0.563	0.0001
Sodium (mg)	366.3	269.8	2911.6	1030.6	299.1	141.6	2237.0	743.7	347.9	218.6	2596.3	1015.8	339.3	270.5	2926.5	1023.3	439.3	274.4	3149.3	933.1	0.284	0.212
Iodine (ug)	21.7	18.5	120.9	68.1	27.9	13.9	104.4 _a_	37.8	31.6	18.6	117.1 _a_	53.4	21.1	18.9	118.2 _a_	53.7	18.1	15.9	130.3 _b_	84.7	0.144	0.0003
Manganese (mg)	0.7	0.9	2.7	1.7	0.5	0.7	2.0	1.2	0.7	1.0	2.4	1.6	0.7	0.9	2.6	1.4	0.9	1.0	3.0	2.0	0.372	0.254
Phosphorus (mg)	196.8	140.9	1163.1	374.5	253.9	120.6	1042.4 _a_	296.4	287.4	150.9	1199.7 _a_	445.6	191.8	153.9	1175.7 _a_	399.3	167.9	98.4	1166.9 _a_	289.8	0.070	0.038
Potassium (mg)	591.3	311.3	2751.3	903.2	551.1	227.1	2199.8 _a_	620.7	638.4	295.1	2489.5 _a_	847.5	582.6	318.8	2746.2 _b_	929.7	609.7	289.4	2987.0 _c_	748.0	0.237	0.002
Magnesium (mg)	68.0	45.9	298.9	108.6	61.7	31.3	233.4 _a_	68.2	70.5	40.0	267.1 _a_	96.8	69.4	52.0	305.5 _b_	115.1	69.6	40.2	317.8 _c_	93.6	0.103	0.002
Copper (mg)	0.6	0.6	2.0	1.5	0.2 ^a^	0.1	1.1 _a_	0.6	0.2 ^a^	0.2	1.2 _a_	1.0	0.6 ^b^	0.5	2.0 _b_	1.4	0.7 ^c^	0.5	2.4 _c_	1.5	0.0003	0.027
Selenium (ug)	13.3	9.6	81.7	36.9	6.1 ^a^	3.8	57.6 _a_	23.5	6.9 ^a^	5.0	65.3 _a_	27.3	13.0 ^b^	9.7	81.3 _b_	36.0	17.2 ^c^	8.3	91.5 _c_	33.1	<0.0001	<0.0001

On a given line, different letters indicate significant differences between age groups according to the post-hoc test: superscripts for breakfast data comparisons, subscripts for total day data comparisons.

**Table 3:** Contribution (%) of food groups to intakes of key nutrients at breakfast in children and adolescents (aged 6–17 years) and adults (aged 18 years and over).

	% of Consumers	Energy	Fiber	CHO	Simple Sugars	Free Sugars	Added Sugars	Proteins	Fat	SFA	MUFA	PUFA	B1	B2	B3	B5	B6	B9	B12	Vitamin E	Vitamin D	Vitamin C	Vitamin A	Calcium	Iron	Zinc	Sodium	Manganese	phosphorus	potassium	Magnesium	Copper	Selenium
**Children & Adolescents (6–17 years)**																																	
Milk	80.2	21.2	0.0	14.4	24.1	0.0	0.0	50.4	24.2	29.9	24.4	9.2	18.9	50.6	4.4	35.6	18.4	7.6	60.5	3.9	9.9	0.5	50.0	61.7	2.5	46.9	22.9	1.0	57.9	47.6	35.5	8.2	24.9
Hot Beverages	53.8	7.7	20.1	10.4	13.6	19.7	25.2	4.6	2.9	3.2	2.5	1.5	10.1	3.0	11.8	3.3	10.3	9.2	0.2	18.3	0.0	0.4	0.3	3.5	27.7	4.5	1.3	8.0	6.1	11.3	14.9	16.7	13.0
Fruit Juices	49.3	6.0	6.1	9.0	15.1	22.1	0.4	2.8	0.4	0.2	0.4	2.4	8.9	2.2	6.3	5.6	6.9	19.0	0.0	5.1	0.8	72.7	6.3	6.0	1.4	1.1	0.7	6.0	2.4	14.0	7.8	6.2	9.6
Breads & toasts	55.3	11.3	24.7	14.4	1.5	0.8	1.0	12.3	3.8	2.1	3.5	11.7	5.6	1.9	8.3	3.9	3.7	6.1	2.8	3.1	3.4	0.0	0.2	3.1	14.7	18.5	30.0	46.7	6.9	4.7	13.3	15.5	8.0
>wholegrain	3.2	0.3	1.1	0.4	0.1	0.0	0.1	0.4	0.1	0.1	0.2	0.5	0.3	0.1	0.7	0.2	0.3	0.3	0.1	0.2	0.1	0.0	0.0	0.2	1.0	1.2	0.8	3.9	0.4	0.2	0.8	1.0	0.3
Breakfast cereals	54.4	14.7	20.3	19.2	13.0	18.4	23.5	7.9	7.2	6.1	7.7	12.5	47.2	31.1	61.1	40.9	51.9	39.1	19.8	19.7	7.5	21.5	0.1	10.5	33.0	9.4	14.3	13.2	7.2	7.4	9.3	13.9	9.0
>wholegrain	27.8	6.1	9.5	8.0	5.5	8.0	10.2	3.2	2.9	2.9	3.0	3.9	19.7	13.0	25.6	16.6	21.8	16.5	8.3	5.4	1.7	11.7	0.0	4.5	14.0	4.1	5.9	6.8	3.1	3.6	4.3	5.9	6.1
Viennoiseries	49.4	12.3	6.7	9.5	3.3	4.4	5.7	8.6	20.2	18.5	15.6	16.9	3.1	2.6	3.3	4.2	2.3	9.8	4.3	11.6	19.0	0.1	11.7	3.8	6.0	5.8	17.2	8.0	4.5	3.3	3.9	10.4	11.3
Sugar & Sweets	64.1	8.8	8.4	9.0	14.6	20.5	26.2	3.0	11.0	7.9	17.0	13.5	1.7	2.3	1.8	1.4	2.2	1.8	2.1	18.3	1.8	0.4	0.6	2.0	6.4	3.5	1.1	7.8	3.2	4.0	6.2	11.1	3.3
Fresh Dairy	13.1	1.4	0.1	1.3	2.0	1.0	1.3	2.1	1.3	1.6	1.0	0.4	0.4	1.8	0.2	0.9	0.4	1.1	2.1	0.2	5.3	0.1	1.2	2.5	0.2	1.7	0.9	0.3	2.0	1.3	0.8	0.5	1.6
Fats & Oils	32.7	2.7	0.0	0.0	0.0	0.0	0.0	0.1	10.0	12.5	8.0	7.2	0.1	0.1	0.0	0.1	0.0	0.1	0.2	4.9	6.3	0.0	14.3	0.1	0.1	0.2	1.0	0.3	0.1	0.1	0.1	0.9	0.7
Fruit	9.1	0.4	2.2	0.6	1.0	0.0	0.0	0.2	0.1	0.0	0.0	0.3	0.3	0.2	0.3	0.4	0.9	1.2	0.0	1.0	0.0	2.7	0.7	0.1	0.2	0.2	0.1	1.2	0.2	1.2	0.7	1.2	0.8
Total		86.4	88.5	87.8	88.2	87.0	83.3	92.0	80.8	81.9	80.1	75.5	96.1	95.8	97.5	96.3	97.2	94.9	92.1	85.9	54.0	98.2	85.4	93.6	92.1	91.6	89.5	92.4	90.6	94.8	92.5	84.6	82.1
**Adults (18 years +)**																																	
Milk	48.6	10.6	0.0	7.1	14.9	0.0	0.0	28.2	11.7	14.1	13.4	3.8	15.4	39.2	2.5	27.7	16.2	4.9	49.7	2.3	4.1	0.4	20.7	41.2	1.8	25.7	8.8	0.3	38.8	21.6	15.1	1.1	5.0
Hot Beverages	89.4	4.3	11.7	4.6	6.2	7.3	9.2	6.3	2.7	2.1	2.2	1.4	8.8	17.6	39.5	16.7	6.2	6.6	2.2	7.7	0.1	0.6	1.8	19.6	18.2	11.5	2.5	20.3	8.5	41.1	41.6	75.6	67.5
Fruit Juices	33.1	4.5	3.9	6.7	13.9	20.2	0.4	2.5	0.3	0.1	0.4	1.5	11.2	2.6	5.3	6.9	8.9	20.3	0.0	5.5	0.3	68.9	6.3	6.1	1.6	1.0	0.5	4.0	2.7	10.0	5.4	1.5	3.0
Bread & Toasts	68.5	29.2	49.2	37.6	4.3	1.3	1.6	34.4	7.8	3.6	7.7	23.8	21.6	6.5	19.5	13.1	16.9	21.8	9.0	10.0	11.9	0.1	0.3	10.0	38.8	36.7	58.4	54.3	21.7	10.3	21.0	8.8	7.6
>wholegrain	8.6	1.7	5.0	2.0	0.4	0.3	0.4	2.1	0.8	0.4	1.0	2.1	1.7	0.6	3.4	1.2	2.2	1.3	0.3	1.2	0.2	0.0	0.0	1.0	6.8	6.6	3.1	13.8	2.3	0.9	3.5	1.2	0.7
Breakfast cereals	16.0	4.9	7.4	6.2	4.6	6.5	8.1	3.5	2.6	2.3	3.0	4.0	26.6	16.8	24.0	18.8	31.8	17.6	11.2	7.0	3.8	10.1	0.0	3.4	16.8	4.3	4.9	4.8	3.5	2.1	3.0	2.6	1.3
>wholegrain	10.5	3.1	5.2	3.7	2.8	4.0	5.0	2.3	2.0	1.9	2.2	2.8	16.8	10.6	15.1	10.6	20.1	11.1	7.1	4.5	1.2	7.3	0.0	1.8	11.0	3.2	3.2	3.7	2.5	1.5	2.1	1.4	1.1
Viennoiseries	37.2	11.2	6.2	8.5	3.5	4.6	5.7	8.4	18.6	17.7	15.8	12.1	4.7	3.5	3.0	5.2	3.6	11.6	5.7	12.3	22.0	0.1	9.2	4.3	7.1	5.6	11.9	5.2	5.2	2.5	2.9	2.5	3.4
Sugar & Sweets	76.2	11.7	5.5	17.3	36.1	49.1	61.3	1.5	2.8	2.1	4.5	3.1	1.1	1.4	1.1	1.1	2.5	1.3	0.8	5.4	0.2	1.4	0.8	1.4	3.9	1.6	0.5	1.8	1.5	2.1	2.0	1.5	0.5
Fresh Dairy	12.1	1.6	0.2	1.1	2.1	1.3	1.7	4.2	1.9	2.2	1.7	0.6	1.4	4.6	0.4	2.8	1.6	3.3	6.2	0.6	6.5	0.2	1.9	5.2	0.5	3.0	1.0	0.2	4.3	1.8	1.2	0.2	1.0
Fats & Oils	51.6	9.2	0.0	0.1	0.1	0.0	0.0	0.5	33.8	39.5	31.6	26.8	0.7	0.4	0.1	0.4	0.2	0.3	1.4	28.2	19.6	0.1	40.3	0.4	0.6	0.8	2.7	1.0	0.5	0.2	0.2	1.3	1.1
Fruit	14.3	1.7	7.7	2.4	4.7	0.0	0.0	1.0	0.3	0.1	0.1	1.2	2.1	1.1	1.2	2.3	5.7	5.6	0.0	5.7	0.0	13.7	2.7	0.9	1.0	0.9	0.3	2.6	1.2	3.8	2.2	1.3	1.3
Total		89.0	91.7	91.6	90.5	90.3	87.8	90.6	82.4	83.8	80.6	78.3	93.5	93.5	96.5	95.1	93.7	93.2	86.3	84.7	68.6	95.5	84.0	92.3	90.3	91.0	91.6	94.7	87.8	95.6	94.5	96.4	91.6

CHO: Carbohydrates; SFA: Saturated fatty acids; MUFA: Monounsaturated fatty acids; PUFA: Polyunsaturated fatty acids.

**Table 4:** Mean (SD) intake of nutrients at breakfast across tertiles of NRF 9.3 score by age group (all breakfast days included).

	Children and Adolescents (6–17 years)						Adults (18 years +)					
	T1 (*n* = 231; *nw* = 221)	T2 (*n* = 213; *nw* = 224)	T3 (*n* = 222; *nw* = 234)	Model A *	Model B *		T1 (*n* = 322; *nw* = 332)	T2 (*n* = 338; *nw* = 338)	T3 (*n* = 364; *nw* = 351)	Model A **	Model C **	
				*p*-Value	*p*-Value Model	*p*-Value for Tertiles Effect				*p*-Value for Tertiles Effect	*p*-Value Model	*p*-Value for Tertiles Effect
NRF 9.3	558.1 (45.9) ^a^	638.6 (17.2) ^b^	713.9 (35.9) ^c^	<0.001	0.001	<0.001	579.3 (55.2) ^a^	677.7 (18.9) ^b^	757.6 (33.5) ^c^	<0.001	<0.001	<0.001
Quantity (g)	320 (116.1) ^a^	361.3 (111.5) ^b^	365.2 (121.1) ^b^	<0.001	0.048	<0.001	413.4 (180.5) ^a^	452.0 (203.2) ^b^	492.0 (173.8) ^c^	<0.001	<0.001	0.001
Energy (kcal)	365 (144.7)	387.5 (132.7)	383.8 (153.9)	0.213	0.048	0.103	325.8 (203.2)	343.0 (181.6)	343.2 (157.9)	0.356	0.231	0.377
Energy wa (kcal)	365 (144.7)	387.4 (132.7)	383.7 (153.9)	0.213	0.001	0.103	325.7 (203.2)	343.0 (181.7)	343.2 (157.9)	0.357	0.233	0.378
Dietary fiber (g)	2.2 (1.3) ^a^	2.5 (1.2) ^b^	2.8 (1.8) ^c^	<0.001	0.036	<0.001	2.0 (1.7) ^a^	2.5 (1.9) ^b^	3.3 (2.1) ^c^	<0.001	<0.001	<0.001
Carbohydrates (g)	55.1 (21.6) ^a^	59.7 (21.7) ^b^	59.7 (25.8) ^b^	0.053	0.760	0.026	49.0 (32.0)	53.8 (30.6)	54.6 (26.5)	0.028	0.166	0.059
Carbohydrates (% energy wa)	61.4 (10.0)	61.8 (8.6)	61.8 (10.9)	0.857	0.087	0.904	60.7 (17.8)	62.3 (14.0)	63.6 (13.0)	0.043	0.090	0.172
Simple sugars (g)	32.4 (13.4)	33.8 (12.2)	32.9 (14.2)	0.537	0.397	0.334	23.4 (16.1)	24.0 (15.5)	24.7 (14.7)	0.525	<0.001	0.083
Free sugars (g)	22.8 (11.4)	23.2 (10.5)	21.6 (11.5)	0.296	0.121	0.343	17.1 (12.2)	16.9 (12.5)	15.4 (10.9)	0.107	<0.001	0.346
Free sugars (% energy wa)	26.2 (12.4) ^a^	24.4 (9.1) ^a,b^	22.8 (10.7) ^b^	0.003	0.031	0.002	26.4 (23.1) ^a^	21.9 (16.0) ^b^	19.5 (14.8) ^b^	<0.001	<0.001	0.001
Added sugars (g)	18.3 (10.6)	18.1 (9.5)	16.4 (9.8)	0.087	0.002	0.225	14.2 (10.9)	13.0 (9.6)	12.4 (10.2)	0.066	0.022	0.253
Added sugars (% energy wa)	19.7 (7.9) ^a^	18.5 (6.8) ^a^	16.7 (7.3) ^b^	<0.001	0.001	<0.001	22.5 (22.4) ^a^	17.1 (13.9) ^b^	16.0 (14.6) ^b^	<0.001	<0.001	<0.001
Proteins (g)	10.3 (5.1) ^a^	11.5 (4.7) ^b^	11.8 (5.0) ^b^	0.004	0.006	<0.001	8.3 (6.1) ^a^	9.1 (5.6) ^b^	9.9 (5.5) ^c^	0.002	0.036	0.001
Proteins (% energy wa)	11.2 (3.6) ^a^	11.9 (3.1) ^b^	12.4 (3.2) ^b^	<0.001	0.142	<0.001	12.2 (13.8)	11.9 (10.2)	12.2 (8.0)	0.879	0.626	0.796
Fat (g)	11.5 (6.3)	11.4 (6.0)	10.8 (5.8)	0.439	0.520	0.578	10.7 (9.0)	10.1 (6.6)	9.5 (6.5)	0.077	0.015	0.134
Fat (% energy wa)	27.5 (9.4)	26.3 (8.6)	25.8 (10.4)	0.149	0.328	0.131	27.1 (13.7) ^a^	25.8 (11.6) ^a,b^	24.2 (10.7) ^b^	0.008	0.024	0.037
SFA (g)	5.4 (2.9)	5.6 (3.2)	5.4 (3.0)	0.855	0.690	0.793	5.5 (5.9) ^a^	5.1 (3.7) ^a^	4.5 (3.5) ^b^	0.013	0.015	0.015
SFA (% energy wa)	13.1 (5.0)	12.9 (4.6)	13.2 (6.6)	0.830	0.091	0.855	13.6 (8.6) ^a^	13.0 (7.2) ^a^	11.5 (6.3) ^b^	0.001	0.011	0.002
MUFA (g)	3.7 (2.3)	3.6 (2)	3.3 (1.8)	0.078	0.203	0.209	3 (2.3)	2.8 (1.9)	2.7 (1.9)	0.131	0.001	0.635
MUFA (% energy wa)	8.7 (3.2) ^a^	8.3 (3.1) ^a^	7.8 (3.1) ^b^	0.005	0.043	0.009	7.7 (4.0)	7.2 (3.4)	6.9 (3.4)	0.016	<0.001	0.433
PUFA (g)	1.1 (0.8)	1.0 (0.7)	1.0 (0.7)	0.587	0.127	0.713	1.0 (1.0) ^a^	1.0 (0.9) ^a^	1.2 (1.2) ^b^	0.008	0.001	0.028
PUFA (% energy wa)	2.5 (1.3)	2.3 (1.3)	2.2 (1.1)	0.075	0.001	0.079	2.5 (2.1) ^a^	2.5 (1.7) ^a^	3.1 (2.2) ^b^	<0.001	0.009	0.002
Water (g)	237.8 (94.1) ^a^	272.6 (92.1) ^b^	276.2 (97.6) ^b^	<0.001	0.037	<0.001	339.9 (162.7) ^a^	372.5 (188.0) ^b^	410.5 (160.0) ^c^	<0.001	<0.001	0.002
Vitamin B1 (mg)	0.3 (0.2) ^a^	0.4 (0.3) ^b^	0.4 (0.3) ^b^	0.006	0.015	0.001	0.22 (0.21) ^a^	0.19 (0.2) ^a^	0.19 (0.21) ^b^	0.002	<0.001	<0.001
Vitamin B2 (mg)	0.5 (0.3) ^a^	0.6 (0.4) ^b^	0.6 (0.4) ^b^	0.006	0.039	<0.001	0.3 (0.3) ^a^	0.3 (0.3) ^a^	0.4 (0.4) ^b^	0.002	<0.001	<0.001
Vitamin B3 (mg)	3.0 (2.5) ^a^	3.6 (3.3) ^b^	3.8 (3.2) ^b^	0.007	0.062	0.002	2.5 (2.9) ^a^	2.6 (2.5) ^a^	3.3 (3.2) ^b^	<0.001	<0.001	<0.001
Vitamin B5 (mg)	1.4 (1.0) ^a^	1.7 (1.2) ^b^	1.7 (1.1) ^b^	0.007	0.061	0.002	0.9 (0.9) ^a^	0.9 (0.7) ^a^	1.0 (0.8) ^b^	0.036	<0.001	<0.001
Vitamin B6 (mg)	0.4 (0.3) ^a^	0.5 (0.4) ^b^	0.5 (0.4) ^b^	0.009	0.042	0.002	0.2 (0.3) ^a^	0.2 (0.2) ^a^	0.3 (0.3) ^b^	<0.001	<0.001	<0.001
Vitamin B9 (ug)	55.8 (30.1) ^a^	65.9 (40.0) ^b^	66.5 (40.5) ^b^	0.003	0.019	0.001	38.2 (36.5) ^a^	44.8 (34.9) ^b^	49.3 (38.9) ^b^	<0.001	<0.001	<0.001
Vitamin B12 (ug)	0.5 (0.4) ^a^	0.6 (0.4) ^b^	0.6 (0.4) ^b^	0.010	0.088	0.001	0.3 (0.4) ^a^	0.3 (0.3) ^a^	0.4 (0.4) ^b^	0.002	<0.001	<0.001
Vitamin E (mg)	1.6 (1.2)	1.4 (1.1)	1.3 (0.9)	0.037	0.851	0.228	0.9 (1.0) ^a^	1.0 (1.0) ^a^	1.1 (1.1) ^b^	0.029	0.025	0.017
Vitamin D (ug)	0.2 (0.3)	0.2 (0.3)	0.2 (0.4)	0.403	0.080	0.358	0.2 (0.3)	0.2 (0.3)	0.2 (0.3)	0.713	0.124	0.390
Vitamin C (mg)	21.0 (25.0) ^a^	26.1 (27.9) ^a^	28.3 (31.6) ^b^	0.020	0.018	0.049	15.1 (27.5) ^a^	20.8 (31.7) ^b^	21.8 (25.2) ^b^	0.004	<0.001	<0.001
Vitamin A (ug)	68.0 (40.3) ^a^	77.3 (47.9) ^b^	80 (51.4) ^b^	0.016	0.012	0.003	75.6 (76.4)	80.3 (86.3)	81.6 (82.0)	0.612	0.047	0.765
Retinol (ug)	56.3 (36.2) ^a^	65.7 (43.4) ^b^	67.9 (46.2) ^b^	0.008	0.441	0.002	64.9 (70.1)	64.4 (57.0)	64.6 (73.0)	0.997	0.016	0.845
Beta-carotene (ug)	131.9 (234.7)	120.8 (193.9)	132 (210.7)	0.815	0.001	0.903	106.0 (228.3)	133.1 (424.3)	138.9 (260.0)	0.352	0.062	0.128
Calcium (mg)	272.1 (148.0) ^a^	312.2 (152) ^b^	320.1 (141.4) ^b^	0.001	0.011	<0.001	187.2 (158.8) ^a^	202.2 (138.2) ^a^	227.8 (145.1) ^b^	0.001	<0.001	<0.001
Iron (mg)	2.9 (1.8) ^a^	3.3 (2.0) ^b^	3.5 (2.2) ^b^	0.014	0.010	0.005	1.7 (1.7) ^a^	1.8 (1.6) ^a^	2.5 (2.2) ^b^	<0.001	<0.001	<0.001
Zinc (mg)	1.3 (0.7) ^a^	1.4 (0.7) ^b^	1.6 (0.9) ^c^	<0.001	0.037	<0.001	1.0 (0.8) ^a^	1.1 (0.8) ^b^	1.5 (1.1) ^b^	<0.001	<0.001	<0.001
Sodium (mg)	290.4 (150.1) ^a^	329.5 (166.9) ^b^	350.3 (193.5) ^b^	0.001	0.008	0.001	346.2 (299.8)	404.4 (283.9)	400.3 (236.5)	0.009	0.001	0.156
Iodine (ug)	27.8 (15.9) ^a^	30.3 (14.8) ^a,b^	31.3 (15.9) ^b^	0.056	0.020	0.004	19 (16.1) ^a^	20.1 (18.8) ^b^	21.7 (17.8) ^c^	0.135	<0.001	0.004
Manganese (mg)	0.5 (0.6) ^a^	0.6 (0.7) ^a^	0.8 (1.1) ^b^	0.002	0.001	0.025	0.5 (0.6) ^a^	0.7 (0.8) ^a^	1.1 (1.2) ^b^	<0.001	<0.001	<0.001
Phosphorus (mg)	250.5 (135.8) ^a^	283.3 (124.9) ^b^	280.5 (127.9) ^b^	0.013	<0.001	0.001	176.4 (140.8) ^a^	178.9 (124.3) ^a^	200.2 (131.5) ^b^	0.035	<0.001	<0.001
Potassium (mg)	541.1 (251.9) ^a^	614.4 (234.4) ^b^	629.6 (252.7) ^b^	<0.001	0.007	<0.001	551.3 (280.8) ^a^	588.1 (307.2) ^a^	664.6 (299.8) ^b^	<0.001	0.001	<0.001
Magnesium (mg)	59.8 (33.6) ^a^	65.5 (28.4) ^b^	73.2 (38.7) ^c^	<0.001	0.225	<0.001	61.6 (46) ^a^	67.2 (41.8) ^a^	82.3 (49.7) ^b^	<0.001	<0.001	<0.001
Copper (mg)	0.2 (0.1)	0.2 (0.2)	0.2 (0.2)	0.052	0.878	0.107	0.6 (0.5)	0.6 (0.6)	0.7 (0.5)	0.415	<0.001	0.762
Selenium (ug)	6.3 (3.8)	6.6 (4.3)	6.7 (4.7)	0.578	<0.001	0.773	13.8 (9.3)	14.4 (9.7)	16.2 (8.7)	0.002	<0.001	0.342

*n*: sample size; *nw*: weighted sample size; SFA: Saturated fatty acids; MUFA: Monounsaturated fatty acids; PUFA: Polyunsaturated fatty acids; wa: without alcohol; Analyis based on breakfast consumption days only; On a given line, different superscript letters indicate significant differences between tertiles of same age group, according to post-hoc test; * For children and adolescents, Model A: One-way ANOVA, without adjustment; Model B: ANCOVA, adjustment for head of household occupational status and head of household education; ** For adults, Model A: One-way ANOVA, without adjustment; Model C: Model B + adjustment for age (continuous).

**Table 5:** Food intakes at breakfast (mean (SD)) and % of consumers across tertiles of NRF 9.3 score by age group (all breakfast days included).

	Children (6–17 years)						Adults (18 years +)					
	T1 (*n* = 231; *nw* = 221)	T2 (*n* = 213; *nw* = 224)	T3 (*n* = 222; *nw* = 234)	Model A *	Model B **		T1 (*n* = 322; *nw* = 332)	T2 (*n* = 338; *nw* = 338)	T3 (*n* = 364; *nw* = 351)	Model A *	Model C ***	
				*p*-Value	*p*-Value Model	*p*-Value Tertiles Effect				*p*-Value	*p*-Value Model	*p*-Value Tertiles Effect
NRF 9.3	558.1 ^a^ (45.9)	638.6 ^b^ (17.2)	713.9 ^c^ (35.9)	<0.001	0.001	<0.001	579.3 ^a^ (55.2)	677.7 ^b^ (18.9)	757.6 ^c^ (33.5)	<0.001	<0.001	<0.001
Milk (mL)	154.6 ^a^ (127.9) 75.8%	179.6 ^b^ (128.0) 83.2%	180.1 ^b^ (122.1) 85.0%	0.051	0.002	0.003	78.7 ^a^ (117.5) 48.4%	71.6 ^a^ (98.6) 47.9%	83.9 ^b^ (108.4) 51.7%	0.331	0.002	0.035
Hot Beverages (mL)	18.4 (39.7) 47.3%	27.2 (61.3) 60.2%	29.6 (67.7) 55.9%	0.091	0.083	0.200	204.2 (177.5) 86.7%	213.4 (179.5) 91.6%	237.6 (157.4) 93.9%	0.031	0.083	0.573
Fruit Juices (mL)	49.3 (72.2) 44.8%	55.6 (75.0) 51.2%	58.2 (81.5) 53.8%	0.438	0.453	0.558	33.1 ^a^ (65.3) 29.2%	46.4 ^b^ (82.9) 37.1%	33.8 ^a,b^ (54.2) 34.5%	0.017	0.453	0.005
Breads & toasts (g)	11.1 ^a^ (20.1) 44.1%	14.9 ^a^ (23.5) 61.9%	19.8 ^b^ (28.2) 61.8%	0.001	0.001	0.005	26.0 ^a^ (37.0) 56.2%	37.2 ^b^ (40.7) 70.9%	38.7 ^b^ (33.9) 80.8%	<0.001	0.001	0.046
>wholegrain	0.2 (1.7) 1.6%	0.2 (1.5) 2.7%	1.0 (5.0) 5.2%	0.013	0.036	0.157	0.8 ^a^ (5.7) 4.1%	1.2 ^a^ (5.5) 6.4%	4.2 ^b^ (14.6) 15.5%	<0.001	0.036	<0.001
Breakfast cereals (g)	11.2 ^a^ (17.6) 48.1%	15.8 ^b^ (22.5) 61.2%	15.5 ^b^ (21.3) 56.2%	0.028	0.201	0.021	4.1 ^a^ (14.5) 16.3%	3.1 ^a^ (11.6) 12.7%	7.4 ^b^ (19.4) 19.6%	0.001	0.201	<0.001
>wholegrain	3.7 ^a^ (9.0) 23.3%	7.2 ^b^ (13.7) 33.0%	6.8 ^b^ (16.0) 28.4% ^b^	0.009	0.147	0.021	2.2 ^a^ (9.7) 9.6%	1.7 ^a^ (9.0) 7.5%	5.5 ^b^ (17.1) 14.9%	<0.001	0.147	<0.001
Viennoiseries (g)	13.8 (18.1) 56.0%	10.6 (16.4) 51.5%	10.6 (18.6) 43.4%	0.080	0.485	0.129	11.4 ^a^ (21.5) 41.4%	11.0 ^a^ (19.9) 40.7%	6.2 ^b^ (12.4) 31.4%	<0.001	0.485	0.003
Sugar & Sweets (g)	8.8 (13.1) 59.7%	8.4 (11.6) 68.7%	6.9 (9.5) 66.7%	0.174	0.844	0.077	12.6 (15.6) 78.7%	13.0 (14.0) 77.7%	13.3 (16.4) 75.9%	0.806	0.844	0.313
Fresh Dairy (g)	7.0 (21.7) 14.7%	5.2 (19.0) 10.7%	7.2 (22.9) 14.4%	0.548	0.116	0.663	2.8 ^a^ (14.5) 5.4%	8.5 (27.7) 12.1% ^b^	12.9 ^b^ (32.9) 19.0%	<0.001	0.116	0.001
Fats & Oils (g)	1.1 ^a^ (3.4) 24.0%	1.5 ^a,b^ (3.1) 37.3%	1.9 ^b^ (3.6) 38.0%	0.060	0.032	0.062	4.0 (6.9) 42.4%	5.3 (7.3) 54.7%	5.5 (7.0) 59.8%	0.016	0.032	0.724
Fruit (g)	1.5 (8.8) 5.4%	2.7 (10.7) 8.9%	4.4 (14.0) 13.3%	0.027	0.333	0.064	3.2 ^a^ (19.4) 5.8%	8.0 ^a^ (27.2) 13.3%	18.8 ^b^ (44.9) 24.0%	<0.001	0.333	<0.001

*n*: sample size; *nw*: weighted sample size; Analysis based on breakfast consumption days only, among breakfast consumers only. On a given line, different superscript letters indicate significant differences between tertiles of same age group, according to post-hoc test; * Model A: One-way ANOVA, without adjustment; ** Model B: ANCOVA, adjustment for head of household occupational status and head of household education; *** Model C: Model B + adjustment for age (continuous).

## Appendix A. Governing Principles Document

You, __________________________________ (name of contributor), have been engaged by CPW SA based on your topic-expert credentials to participate in research work with the aim to establish a multi-disciplinary expert recommendation on the nutrient and food criteria of a healthy breakfast for European children and adults (the “Project”).

The Project is being structured and these governing principles are being issued to ensure the scientific integrity of the Project research work and the resulting publication.

These principles supplement the separate agreement(s) that you have or will enter into to govern your participation in the Project. We kindly ask you to review them carefully and to confirm below that you understand and agree to them.

We, as CPW SA, expect the following governing principles to be respected and adhered to by all participants in the Project for the duration of the Project:

- Participants are expected to provide impartial and objective input throughout the Project.
- Participants are under no obligation to promote or communicate about CPW SA, its products or the breakfast cereal category.
- To the extent a participant makes public statements or other comments (e.g., on social media) regarding CPW SA or its products, he/she shall disclose his/her material connection to the Company.
- The Steering Committee will decide on authorship and intellectual input into the scientific paper, but it is anticipated that the paper will be drafted by the Project Chair and Co-Chair with iterative inputs from the other participants. The Project Chair will submit the final paper for publication.
- The resulting publication shall accurately reflect the funding by CPW of the Project. In case a CPW SA employee materially participates in the drafting of the paper, this shall also be accurately reflected.

By signing below, you agree that you understand and agree to be bound by the above governing principles.

Signature: ______________________________

Name: ______________________________

Place and date: ______________________________

**Table A1:** Consumption of solid foods (grams) and beverages (mL) at breakfast in four age groups.

	Children		Adolescents		Younger Adults		Older Adults		ANCOVA *
	(6–12 years)		(13–17 years)		(18–54 years)		(55 years and above)		
	*N* = 405		*N* = 285		*N* = 634		*N* = 403		
Solid Foods	Mean	SD	Mean	SD	Mean	SD	Mean	SD	*p*-value
Sweet crackers and biscuits	6.1 ^a^	0.8	5.3 ^a^	0.9	5.0 ^a^	0.6	0.8 ^b^	0.2	<0.001
Of which wholegrain	0.9	0.2	0.2	0.1	0.6	0.3	0.1	0	0.054
Of which non-wholegrain	5.2 ^a^	0.7	5.1 ^a^	0.9	4.4 ^a^	0.5	0.8 ^b^	0.2	<0.001
Breakfast cereals	13.5 ^a^	0.8	14.7 ^a^	1.5	6.4 ^b^	0.7	2.3 ^c^	0.6	<0.001
Of which wholegrain	5.2 ^a,b^	0.5	6.7 ^a^	1	3.9 ^b,c^	0.5	2.0 ^c^	0.5	<0.001
Of which non-wholegrain	8.3 ^a^	0.7	8.0 ^a^	1.2	2.5 ^b^	0.4	0.4 ^c^	0.2	<0.001
Fruit desserts	0.8	0.2	1.1	0.4	1.4	0.5	1.3	0.4	0.67
Milk desserts	0.3	0.1	0.4	0.3	0.3	0.1	0.5	0.2	0.967
Fruits	3.0 ^a^	0.5	2.7 ^a^	0.7	9.0 ^a^	1.2	11.7 ^b^	1.8	<0.001
Fats and oils	1.6 ^a^	0.1	1.4 ^a^	0.2	3.2 ^b^	0.2	7.4 ^c^	0.4	<0.001
Breads and toasts	13.3 ^a^	1	18.4 ^a^	1.9	25.3 ^b^	1.4	46.4 ^c^	1.9	<0.001
Of which wholegrain	0.5 ^a^	0.2	0.5 ^a^	0.2	1.7 ^b,c^	0.3	2.6 ^c^	0.6	<0.001
Of which non-wholegrain	12.8 ^a^	1	18.0 ^a^	1.9	23.6 ^b^	1.4	43.8 ^c^	1.8	<0.001
Cakes and pies	3.8 ^a^	0.4	4.0 ^a^	0.7	3.1 ^a^	0.5	1.4 ^b^	0.4	0.001
Sugars and sweets	7.2 ^a^	0.5	9.2 ^a,b^	0.9	10.6 ^b^	0.6	16.2 ^c^	0.8	<0.001
Fresh dairy products	7.1 ^a,b^	1.1	5.2 ^a^	1.2	6.4 ^a^	0.9	10.6 ^b^	1.5	0.018
Viennoiseries	10.3 ^a^	0.7	13.3 ^a^	1.3	10.8 ^a^	0.8	6.9 ^b^	0.7	<0.001
Other products (cheese, eggs, meat, …)	0.4 ^a^	0.1	0.8 ^a^	0.2	2.5 ^b^	0.6	4.6 ^c^	0.8	
Beverages									
Sodas	8.5 ^a,b^	1.7	11.3 ^a^	2.9	6.3 ^a,b^	1.3	2.6 ^b^	1	0.005
Milk	164.4 ^a^	5.7	176.0 ^a^	8.6	86.5 ^b^	4.9	61.8 ^c^	4.1	<0.001
Of which milk from hot beverages	68.8 ^a^	4.6	71.6 ^a^	6.4	44.0 ^b^	3.3	52.1 ^b^	3.7	<0.001
Of which milk from other sources	95.5 ^a^	5.5	104.4 ^a^	8.3	42.5 ^b^	4.1	9.7 ^c^	2	<0.001
Water	17.4	2.2	14.4	3	16.2	2.3	19.4	2.7	0.608
Juices and nectars	46.2 ^a^	3.1	63.7 ^b,c^	5.4	43.8 ^c^	3	26.6 ^d^	2.7	<0.001
Hot beverages	20.6 ^a^	2.3	30.9 ^a^	4.2	183.9 ^b^	7	264.9 ^c^	7.8	<0.001

* ANCOVA, adjusted for daily total energy intake. On a given line different letter superscripts indicate significant differences between age groups according to the post-hoc test.

**Table A2:** Mean (SD) NRF 9.3 scores by age and individual characteristics.

	Children (6–17 years)	Adults (18 years+)
	(*n* = 676; *nw* = 690)	(*n* = 1040; *nw* = 1037)
**Total**	634.6 (73.2)	674.7 (82.8)
*p*-value	<0.0001	
**Age group**		
6–12	639.5 (70.8)	
13–17	634.7 (78.3)	
*p*-value	0.3938	
18–24 years		627.1 (79.7) ^a^
25–34		648.2 (87.7) ^a,b,c^
35–44		660.0 (96.1) ^b,c^
45–54		671.7 (81.4) ^c,d^
55–64		693.8 (62.7) ^d,e^
>65		705.0 (68.3) ^e^
*p*-value		<0.0001
**Sex**		
Male	636.6 (71.2)	663.4 (87.4)
Female	638.4 (76.5)	680.7 (78.2)
*p*-value	0.7505	0.0007
**Education of the head of household: highest degree obtained**		
No diploma	613.2 (61.4) ^a^	653.1 (81.2) ^a^
Lower than High School Degree	627.7 (68.1) ^a^	665.9 (82.0) ^a^
Vocational Education	626.0 (67.0) ^a,b^	653.7 (87.3) ^b^
High School	658.9 (68.9) ^b,c^	689.5 (85.7) ^b^
Undergraduate	630.0 (97.5) ^a,b^	686.9 (70.8) ^b^
Graduate	673.1 (70.9) ^c^	685.7 (83.7) ^b^
Other	700.6 (57.9) ^a,c,b^	
Missing	630.0 (35.5) ^a,c,b^	704.3 (91) ^a,b^
*p*-value	<0.0001	0.0009
**Occupation of the head of household**		
Farmer, artisan, trader, business owner	647.8 (92.8) ^a,b^	677.9 (84.6) ^a,b^
Executive, Senior Intellectual Profession, Liberal Profession	665.7 (77.0) ^a^	689.7 (80.2) ^a^
Intermediate occupation	643.2 (87.3) ^b^	681.5 (74.5) ^a,b^
Employee	625.7 (60.9) ^b^	668.2 (85.5) ^a,b^
Worker	622.2 (64.3) ^b^	657.7 (81.6) ^b^
Never employed / Inactive	617.6 (58.3) ^a,b^	646.1 (87.5) ^a^
*p*-value	<0.0001	<0.0001
**Type of household**		
Couple without children		681.7 (73.3) ^a^
Couple with children	637.9 (76.2)	655.2 (85.7) ^b^
Single parent family	636.8 (66.6)	654.6 (93.2) ^b^
Single		691.0 (78.9) ^a^
Other	625.5 (54.3)	640.9 (113.4) ^a,b^
*p*-value	0.9136	<0.0001
**Number of children (<15 years)**		
0	625.5 (54.3)	684.0 (77.1) ^a^
1	652.6 (81.6)	653.8 (84.3) ^b^
2	634.9 (70.7)	660.5 (89.4) ^b^
3 or more	633.5 (72.5)	648.8 (90.8) ^b^
*p*-value	0.0841	<0.0001
**Number of people working in the household**		
0	602.6 (56.1) ^a^	695.7 (71.7) ^a^
1	635.7 (74.0) ^a^	661.5 (84.7) ^b^
2 or more	641.1 (74.2) ^b^	661.4 (86.0) ^b^
*p*-value	0.0315	<0.0001
**Living area**		
Rural	629.0 (64.8) ^a^	670.7 (79.1) ^a,b^
2 to 20,000 inhabitants	630.8 (80.9) ^a,b^	666.2 (74.5) ^a,b^
20 to 100,000 inhabitants	635.2 (73.4) ^a,b^	655.6 (90.2) ^a^
>100,000 inhabitants	640.5 (71.1) ^a,b^	680.1 (87.1) ^b^
Paris	655.1 (82.9) ^b^	680.8 (78.4) ^a,b^
*p*-value	0.0472	0.0220
**Region**		
Paris region	648.6 (84.6)	676.9 (77.3) ^a,b^
North France	634.1 (75.8)	664.2 (80.6) ^a^
South France	636.8 (64.8)	680.5 (86.4) ^b^
*p*-value	0.1807	0.0122
**Food budget by consumption unit**		
First Tertile	643.7 (68.2)	667.5 (89.5)
Second Tertile	639.4 (69.3)	666.2 (82.8)
Third Tertile	632.7 (80.0)	679.1 (77.9)
*p*-value	0.2659	0.0536
**Income per consumption unit**		
Less than 9909 €	644.5 (73.1)	666.3 (87.9)
From 9909 € to 12,958 €	618.9 (55.0)	653.4 (86.1)
From 12,958 € to 18,294 €	624.6 (58.2)	664.5 (81.0)
From 18,294 € to 30,490 €	629.4 (80.2)	674.8 (82.8)
More than 30,490 €	645.0 (73.8)	677.7 (75.9)
Missing	642.1 (77.5)	681.1 (85.3)
*p*-value	0.0834	0.2381
**BMI**		
<18.5	628.0 (76.3)	624.0 (103.2) ^a^
18.5–25	637.2 (72.5)	669.9 (85.6) ^b^
25–30	639.2 (75.1)	679.4 (71.6) ^b^
≥30	669.2 (75.7)	680.7 (81.1) ^b^
*p*-value	0.0799	0.0004
**Daily Screen time**		
Lower	644.4 (73.1)	683.3 (75.4)
Higher	630.5 (73.6)	665.1 (86.2)
*p*-value	0.0134	0.0005
**Physical activity**		
Higher	635.8 (76.5)	676.7 (80.0)
Lower	637.1 (69.4)	666.1 (85.5)
*p*-value	0.7758	0.0404
**Smoking**		
Yes		640.6 (90.8) ^a^
No		686.3 (74.9) ^b^
Missing		632.5 (69.4) ^a,b^
*p*-value		<0.0001

*n*: sample size; *nw*: weighted sample size; Among all individuals; Anovas and post-hoc tests. Different superscript letters indicate significant differences between levels of a variable.

**Table A3:** Mean (SD) NRF 9.3 scores and subscores by NRF 9.3 tertiles and age groups.

	Children & Adolescents (6–17 years)						Adults (18 years+)					
	T1 (*n* = 231; *nw* = 221)	T2 (*n* = 213; *nw* = 224)	T3 (*n* = 222; *nw* = 234)	Model A *	Model B *		T1 (*n* = 322; *nw* = 332)	T2 (*n* = 338; *nw* = 338)	T3 (*n* = 364; *nw* = 351)	Model A **	Model C **	
				*p*-Value	*p*-Value Model	*p*-Value for Tertiles Effect				*p*-Value	*p*-Value Model	*p*-Value for Tertiles Effect
NRF 9.3	558.1 (45.9) ^a^	638.6 (17.2) ^b^	713.9 (35.9) ^c^	<0.001	<0.001	<0.001	579.3 (55.2) ^a^	677.7 (18.9) ^b^	757.6 (33.5) ^c^	<0.001	<0.001	<0.001
Positive subscore	655.8 (45.6) ^a^	714.2 (31.4) ^b^	766.3 (34.1) ^c^	<0.001	<0.001	<0.001	674.8 (49.3) ^a^	750.9 (32.3) ^b^	811.3 (34.2) ^c^	<0.001	<0.001	<0.001
Negative subscore	97.8 (38.3) ^a^	75.6 (27.3) ^b^	52.4 (27.0) ^c^	<0.001	<0.001	<0.001	95.5 (45.0) ^a^	73.2 (28.4) ^b^	53.7 (22.8) ^c^	<0.001	<0.001	<0.001
Calcium subscore	0.934 (0.117) ^a^	0.977 (0.061) ^a^	0.988 (0.039) ^b^	<0.001	<0.001	<0.001	0.922 (0.131) ^a^	0.96 (0.091) ^b^	0.983 (0.05) ^c^	<0.001	<0.001	<0.001
Potassium subscore	0.990 (0.030) ^a^	0.999 (0.007) ^b^	1.000 (0.001) ^b^	<0.001	<0.001	<0.001	0.994 (0.030) ^a^	1.000 (0.003) ^b^	1.000 (0.000) ^b^	<0.001	0.001	<0.001
Protein subscore	1.000 (0.001)	1.000 (0.000)	1.000 (0.000)	0.250	0.947	0.345	1.000 (0.000)	1.000 (0.000)	1.000 (0.000)	.	.	.
Fiber subscore	0.566 (0.089) ^a^	0.631 (0.108) ^b^	0.731 (0.120) ^c^	<0.001	<0.001	<0.001	0.607 (0.122) ^a^	0.725 (0.133) ^b^	0.837 (0.128) ^c^	<0.001	<0.001	<0.001
Iron subscore	0.774 (0.134) ^a^	0.855 (0.128) ^b^	0.881 (0.122) ^b^	<0.001	<0.001	<0.001	0.763 (0.147) ^a^	0.826 (0.139) ^b^	0.912 (0.114) ^c^	<0.001	<0.001	<0.001
Vitamin A subscore	0.555 (0.209) ^a^	0.703 (0.221) ^b^	0.820 (0.200) ^c^	<0.001	<0.001	<0.001	0.657 (0.244) ^a^	0.794 (0.218) ^b^	0.882 (0.170) ^c^	<0.001	<0.001	<0.001
Vitamin D subscore	0.362 (0.144) ^a^	0.391 (0.172) ^a^	0.506 (0.242) ^b^	<0.001	<0.001	<0.001	0.399 (0.185) ^a^	0.502 (0.239) ^b^	0.633 (0.256) ^c^	<0.001	<0.001	<0.001
Vitamin C subscore	0.702 (0.274) ^a^	0.843 (0.210) ^b^	0.928 (0.149) ^c^	<0.001	<0.001	<0.001	0.645 (0.279) ^a^	0.870 (0.195) ^b^	0.952 (0.116) ^c^	<0.001	<0.001	<0.001
Magnesium subscore	0.676 (0.088) ^a^	0.744 (0.091) ^b^	0.809 (0.104) ^c^	<0.001	<0.001	<0.001	0.761 (0.129) ^a^	0.831 (0.119) ^b^	0.914 (0.098) ^c^	<0.001	<0.001	<0.001
Sodium subscore	0.149 (0.160)	0.162 (0.177)	0.166 (0.161)	0.504	0.556	0.582	0.346 (0.253) ^a,b^	0.382 (0.241) ^a^	0.324 (0.210) ^b^	0.005	<0.001	<0.001
Saturated fat subscore	0.212 (0.170) ^a^	0.188 (0.165) ^a^	0.125 (0.143) ^b^	<0.001	<0.001	0.000	0.292 (0.285) ^a^	0.219 (0.184) ^b^	0.144 (0.148) ^c^	<0.001	<0.001	<0.001
Free sugars subscore	0.617 (0.423)^a^	0.406 (0.298) ^b^	0.233 (0.258) ^c^	<0.001	<0.001	<0.001	0.316 (0.467) ^a^	0.131 (0.217) ^b^	0.070 (0.134) ^c^	<0.001	<0.001	<0.001

*n*: sample size; *nw*: weighted sample size; Among breakfast consumers only; * For children Model A: One-way ANOVA, without adjustment; Model B: ANCOVA, adjustment for head of household occupational status and head of household education; ** For adults Model A: One-way ANOVA, without adjustment; Model C: Model B + adjustment for age (continuous); On a given line, different superscript letters indicate significant differences between tertiles of same age group, according to post-hoc tests.
